# Coronavirus pandemic and pregnant mothers

**DOI:** 10.1016/j.amsu.2022.104376

**Published:** 2022-08-18

**Authors:** Rozhan Khezri, Layla Shojaie, Hossein-Ali Nikbakht, Sepideh Jahanian, Mousa Ghelichi-Ghojogh

**Affiliations:** Department of Epidemiology, School of Public Health, Iran University of Medical Sciences, Tehran, Iran; Division of GI/Liver, Department of Medicine, Keck School of Medicine, University of Southern California, Los Angeles, CA, USA; Social Determinants of Health Research Center, Health Research Institute, Babol University of Medical Sciences, Babol, Iran; Department of Cardiovascular Surgery, Mayo Clinic, Rochester, MN, USA; Health Management and Social Development Research Center, Golestan University of Medical Sciences, Gorgan, Iran

Dear Editor-in-Chief

In December 2019, four cases of pneumonia from an unknown cause were reported to the World Health Organization (WHO) in Wuhan, China and since then, SARS-COV-2 has spread rapidly worldwide until eventually on March 12, 2020, WHO identified the outbreak as pandemic. SARS-COV-2 lead to the most lethal pandemic in the past century [[1], [2], [3]]. Until the January 3, 2021, According to the WHO, more than 83322449 people have been infected and 183141212 deaths due to Covid 19 have occurred worldwide [4]. Despite the progress on Covid 19 research, there is a paucity of information on the impact of this disease on pregnant women [5]. The main purpose of infectious disease control is to protect people, especially high-risk groups like pregnant women. Respiratory infections lead to increased morbidity and mortality rate in this group. Bacterial and viral pneumonia during pregnancy are life-threatening to the mother. There is still no evidence to suggest that pregnancy increases the chances of Covid-19 infection [6].

Pregnant women are more susceptible to respiratory diseases. During pregnancy, physiological changes in the cardiovascular and immunity systems predispose the mother to infection and hypoxia. The air is not expired properly and the enlarged uterine leads to decreased functional residual and expiratory volumes. Almost 20% of pregnant women experience congestion and rhinorrhea close to term. These symptoms are due to hyperemia and inflammation of nasopharyngeal mucous and may overlap with Covid 19 symptoms. As a result, a person with Undiagnosed Covid-19 can become a carrier of the disease [9].

In addition, during pregnancy, T cells mediated immunity subsides and leads to vulnerability to pathogens such as viruses, including the Covid-19 virus [7]. In late pregnancy, a decrease in the number and activity of NK and T-cell cells leads to the onset and exacerbation of viral infections. The main route of entry of coronavirus into the lung cells is through ACE2 receptor, which is more expressed in pregnant women and can expose this population to Covid-19(8). As a result, pregnant women, especially in the third trimester, can be considered as one of the most high-risk groups in terms of SARS-COV-2 susceptibility. Due to the public health importance of pregnant mothers and consequently their infants, necessary measures should be taken to prevent Covid-19 infection in this group. For pregnant women who are full term, termination of the pregnancy is recommended. For other pregnant mothers, it is necessary to provide the necessary training and encouragement to follow the health protocols to prevent Covid 19 infection.

The Covid-19 pandemic has caused stress and anxiety in pregnant women leading to subsequent problems such as preeclampsia, depression, increased nausea and vomiting during pregnancy, preterm delivery, low birth weight and low Apgar score [8]. It seems that providing immediate counseling services by telephone, virtual or in-person by mental health experts working in comprehensive health service centers per health protocols to this group play an important role in reducing stress and anxiety and future problems during and after pregnancy. It is worth noting that some pregnant women are worried about the health of their baby and fetus. Their concern is about postpartum care such as breastfeeding and infant care programs (such as monthly growth monitoring and vaccinations) [9]. Therefore, providing a platform to increase the awareness of pregnant mothers about transmission of Covid-19 and providing telephone counseling and virtual training through social networks can reduce anxiety in pregnant mothers.

Due to fears and concerns about the risk of Covid-19 infection, some pregnant mothers may not go for prenatal periodic check-ups. Assuming the hospital environment health care centers are infected, they may become post-term, or on the contrary, some may want to terminate the pregnancy immediately through cesarean section [6]. Providing virtual consultation, training and necessary advice to prevent Covid-19 by doctors and midwives in health care units can be helpful regarding this matter.

The American College of Obstetricians and Gynecologists (ACOG) and the Royal College of Obstetricians (RCOG) have issued guidelines for managing pregnant women. Most pregnant women with Covid-19 infection are either asymptomatic or have a mild illness. In the absence of any obstetric complications, they can be managed conservatively at home similar to non-pregnant patients [10]. Therefore, pregnant women must receive mandatory training on the disease symptoms by health care personnel so that they can contact the doctor or midwife immediately if they notice any clinically suspected symptoms of Covid-19.

Life-saving interventions for infectious diseases, especially Covid-19 are crucial for pregnant women, as with all treatment decisions made during pregnancy. This includes assessing the potential risks and benefits of interventions for the mother and fetus. Because monitoring systems have been developed for the coronavirus (Covid-19), information regarding pregnancy status, maternal and fetal outcomes and any preliminary evidence that can guide management and treatment of pregnant women with coronavirus (Covid-19) to prevent adverse outcomes during and after pregnancy should be reported. However, due to the limited number of studies conducted in the country on the consequences of pregnancy and post-pregnancy in pregnant women with coronavirus (Covid-19), it seems that conducting population-based studies of pregnant mothers with Covid-19 can help identify the adverse outcomes and provides a platform for basic and major interventions to deal with adverse maternal and fetal outcomes.

## Sources of funding

This research did not receive any specific grant from funding agencies in the public, commercial, or not-for-profit sectors.

## Ethical Approval

This is a Commentary study and does not require Ethical Approval.

## Consent

This is a commentary study and does not require Consent.

## Author contribution

RK: Idea design, writing—original draft preparation; MG: writing—review; LSH: editing; HA: editing; SJ: writing—review.

## Registration of research studies

This is a commentary study and does not need registration of research studies.

## Guarantor

I accept the liability of the scientific integrity of the manuscript contents.

## Provenance and peer review

Not commissioned, externally peer reviewed.

## Declaration of competing interest

We have no conflicts of interest to disclose.
